# The Quiet Production of Anæsthesia

**Published:** 1901-12

**Authors:** John Freeman

**Affiliations:** Anæsthetist to the Bristol General Hospital.


### THE QUIET PRODUCTION OF ANÆSTHESIA.

### A CLINICAL LECTURE.

BY

JOHN FREEMAN, F.R.C.S. Edin.,

*Anæsthetist to the Bristol General Hospital.*

To succeed in getting patients into the anæsthetic state quietly many little details have to be attended to. One often hears of patients taking the anæsthetic badly, but a good deal depends upon the method employed by the anæsthetist.

The importance of this quiet production of anæsthesia is that the patient's safety is greater. It is when there is struggling, holding of the breath, or vomiting that alarming symptoms may arise. So many deaths have occurred during the "going under," that this is now recognised as the most dangerous period; and in other instances, where death took place later on during the progress of the operation, it was frequently stated that the patients went under badly.

The aim of the anæsthetist should be to make the patient pass into the state of narcosis as quietly as he would fall into natural sleep, and this can be attained in the majority of cases. With this object in view, anything likely to excite the patient should be avoided, and everything possible done to calm him.

It is necessary to remember that the patient is naturally in an anxious state of mind when the time for an operation comes. As a rule he shows a brave attitude; but when you examine the heart, the palpitation present reveals the excited condition of the nervous system. The hospital patient is not so difficult to deal with as the private one. With the latter, the presence of anxious friends does not tend to calm him; and now and then a friend will act in an actually mischievous way, as once happened to a patient of mine. This lady had sent to her by post on the morning of the operation a cutting from a newspaper: it was a report of the death of a person who had died from chloroform. Of course, great anxiety had been caused by this.

Before commencing the anæsthetic, there are a few preliminaries to be attended to.

It is well to examine the chest (unless it has already been done), to get a knowledge of the condition of the heart and lungs. The patient, owing to the palpitation, often has an idea that his heart is not strong, so it is reassuring to him when he can be told he is quite sound. This examination should be performed quickly; for any prolonged application of the stethoscope in the cardiac region has a tendency to alarm the patient, as he thinks you have discovered something wrong there.

The position of the patient as he lies on the table or bed is of some importance. Since one is going to imitate natural sleep as much as possible, the patient should be arranged as comfortably as circumstances will allow. The pillow nearly always requires placing in position, and in doing this see that the patient's head is not too much flexed, otherwise he will not breathe freely as he goes under.

The hands should next be attended to. You will see that they are invariably kept outside the bedclothes; and a nervous patient keeps them near his face, so as to be ready to catch hold of the inhaler. Always have them placed down by the sides, beneath the bedclothes.

The patient at this time will often ask you to be sure he is "asleep" before the operation is begun. You can assure him he will not feel any pain.

The next question frequently asked by the patient is—How shall he breathe? Whether with his mouth open? or whether he must take deep breaths? My answer is, that I wish him to go to sleep as he usually does, so I do not want him to take deep breaths or to keep his mouth open. The more passive you can make him, the better; any active assistance does more harm than good.

Having by such means as these steadied your patient, ask him to close his eyes and go to sleep. The ordinary patient shuts his eyes, but every now and then opens them to see what is being done. To prevent this I place my left hand over his eyes, and wait until the nervousness has passed off and he is breathing regularly. Two or three minutes spent in this way with a nervous patient will save a good deal of time in the end. The patient, with his eyes closed in this manner, sees nothing of the inhaler being applied and does not know when the anæsthetic is really commenced. Many patients imagine they are having it long before they are, and I encourage them in this mistaken idea by telling them they will soon be asleep. This way of "suggesting" sleep can be made of considerable service, provided the anæsthetist has taken the right steps to allay the nervousness of his patient. Many are calmed by it in a remarkable way, and I have met a few who, to all appearances, were well on the way to sleep before any anæsthetic was used.

(This method of suggestion is not much good for a delirious patient or for young children. With the latter, a good way is to divert their attention, if possible, by asking them to count, say, up to 50.)

Regular breathing having been secured, apply the inhaler cautiously and commence the anæsthetic with a weak vapour, its strength being gradually increased as the patient goes under. The breathing is the best guide for increasing the anæsthetic; any hesitation, or coughing, shows that the vapour is too strong at this stage. It is upon the maintenance of regular breathing that the success will now depend; a weak vapour breathed quietly and regularly is more effectual and safer than a stronger one taken intermittently. At the same time, with the patient breathing properly, it is important to go on steadily increasing the vapour, so as not to unnecessarily delay the going under. One sometimes hears of half an hour or more being spent in getting a patient under, which means that this period of danger has been unduly prolonged.

As I have had opportunities of seeing the mistakes made by beginners, I should like to draw your attention to a few of them.

So far as I have been able to observe, asking the patient to take deep breaths is a frequent source of trouble. It at once prevents any imitation of natural sleep, and it often produces reflex holding of the breath or coughing, because a vapour which can be breathed readily with quiet respiration may not be inhaled so well if deep breaths are taken. Imagine, however, a case where these troubles have not arisen, but the patient goes on rapidly taking deep breaths. Now this forced breathing is hard work; and besides that, in a minute or two a condition of apnœa is produced. For these two reasons the patient takes a rest, which is to a great extent a physiological one. A practical anæsthetist is able to see that this is not at all like respiratory failure from paralysis of the centre; but the beginner, thinking the patient has "stopped breathing," commences shouting to him to go on breathing, slaps his face, or may even apply tongue forceps. Any rough treatment of this kind with a patient only partly unconscious should be carefully avoided, as it is frequently followed by alarming symptoms.

These troubles can be prevented by allowing the patient to breathe naturally and quietly at first.

The too energetic testing of the corneal reflex in the early administration is another mistake. Some beginners, having read about the importance of the corneal reflex and the size of the pupil, commence working at the patient's eye from the moment the anæsthetic is started. Nothing can be more irritating to a person going to sleep than this, and I have frequently seen a patient who was going under quietly, roused by it.

There is not any necessity to open the eye in the early stages, so long as the breathing is satisfactory. We have two signs by which we can know the patient is not under. In the first place, there are tremulous movements of the eyelids to be seen, which continue generally until consciousness is lost; and secondly, if when trying to raise the upper eyelid by gently drawing upon the skin near the edge of the orbit there is any resistance offered by the orbicularis, this shows that the muscular system is not relaxed, and consequently that the patient is not under.

Too early interference with the patient for the purpose of removing dressings, clothing, &c., is another way of causing trouble. From the time the anæsthetic is started, no one should touch the patient until he is under.

I have seen struggling brought about through the patient being unnecessarily held by assistants. As he begins to lose consciousness he often moves his limbs a little, just as a person will in a restless sleep. Energetic bystanders may, because of these movements, seize him. This rouses him; he feels he is being held, and begins struggling. So long as the hands are down under the blankets, slight movements need be taken no notice of.

There are other details of the same kind; but if you will remember the system to work upon you will find, after some practice, that no matter whether the anæsthetic be gas, chloroform, or ether, you will have little difficulty with patients in the going-under period. You will also help to allay their anxiety at a trying time, and you will spare them a certain amount of discomfort and danger.

